# High-quality CMOS compatible n-type SiGe parabolic quantum wells for intersubband photonics at 2.5–5 THz

**DOI:** 10.1515/nanoph-2023-0704

**Published:** 2024-01-15

**Authors:** Elena Campagna, Enrico Talamas Simola, Tommaso Venanzi, Fritz Berkmann, Cedric Corley-Wiciak, Giuseppe Nicotra, Leonetta Baldassarre, Giovanni Capellini, Luciana Di Gaspare, Michele Virgilio, Michele Ortolani, Monica De Seta

**Affiliations:** Dipartimento di Scienze, Università; degli Studi Roma Tre, Viale G. Marconi 446, Roma 00146, Italy; Center for Life Nano & Neuro Science, Istituto Italiano di Tecnologia, Viale Regina Elena 291, 00161 Rome, Italy; Department of Physics, “Sapienza” Università di Roma, Piazzale Aldo Moro 2, 00185 Rome, Italy; IHP-Leibniz Institut für Innovative Mikroelektronik, Im Technologiepark 25, Frankfurt (Oder) 15236, Germany; Istituto per la Microelettronica e Microsistemi (CNR-IMM), VIII Strada 5, Catania 95121, Italy; Dipartimento di Fisica “E. Fermi”, Università; di Pisa, Largo Pontecorvo 3, Pisa 56127, Italy

**Keywords:** heterostructures, germanium, terahertz, strong coupling, parabolic potential

## Abstract

A parabolic potential that confines charge carriers along the growth direction of quantum wells semiconductor systems is characterized by a single resonance frequency, associated to intersubband transitions. Motivated by fascinating quantum optics applications leveraging on this property, we use the technologically relevant SiGe material system to design, grow, and characterize n-type doped parabolic quantum wells realized by continuously grading Ge-rich Si_1−*x*_Ge_*x*_ alloys, deposited on silicon wafers. An extensive structural analysis highlights the capability of the ultra-high-vacuum chemical vapor deposition technique here used to precisely control the quadratic confining potential and the target doping profile. The absorption spectrum, measured by means of Fourier transform infrared spectroscopy, revealed a single peak with a full width at half maximum at low and room temperature of about 2 and 5 meV, respectively, associated to degenerate intersubband transitions. The energy of the absorption resonance scales with the inverse of the well width, covering the 2.5–5 THz spectral range, and is almost independent of temperature and doping, as predicted for a parabolic confining potential. On the basis of these results, we discuss the perspective observation of THz strong light–matter coupling in this silicon compatible material system, leveraging on intersubband transitions embedded in all-semiconductor microcavities.

## 1 Introduction

Intersubband transitions (ISBTs) in quantum wells (QWs) have garnered significant attention due to their relevance across a wide spectrum of applications. These transitions between quantized energy levels within the well have unlocked a wealth of opportunities for designing novel electronic and photonic devices with unprecedented performance. In the realm of infrared and terahertz photonics, they enable the development of efficient detectors, modulators, and emitters [1], [2], [3]. ISBTs are also at the heart of quantum cascade lasers (QCLs), a groundbreaking technology that has revolutionized the generation of coherent mid/far-infrared radiation [4], [5], [6].

Among the quantum well profiles, parabolic quantum wells (PQWs) feature a unique harmonic oscillator-like carrier confinement potential along the growth direction. This results in the formation of a ladder-like energy spectrum within the well, with equally spaced quantized energy levels. Furthermore, in modulation-doped parabolic QWs, i.e., QWs with dopant atoms located outside the parabolic potential region, the electron–electron interaction cancels out and, following Kohn’s theorem, the ISB optical resonance frequency *ω*_0_ becomes independent of the carrier density and of their distribution in the different subbands [7]. Consequently, the absorption spectrum of PQWs is virtually temperature independent. This characteristic can be exploited to overcome the thermal charge fluctuation limitations of THz optoelectronic devices based on, i.e., rectangular QWs [5].

ISBTs in a QW system represent the solid-state equivalent of the ideal two-level atomic system but with the advantage of the tunability of the associated quantum transition by a suitable band engineering. In particular, the transition energy can be tuned at the resonance frequency of an optical cavity, a platform that has been used to explore the strong light–matter coupling regimes [8], [9], [10], [11]. The coupling strength can be further externally tuned by leveraging on doping, field-effect [12], Stark-effect [13], magnetic field [10], [14], or optical-field tunability [15]. Indeed, the rate of energy exchange between light and matter excitations in the cavity, usually expressed in terms of the Rabi frequency Ω_*R*_, can be varied acting on the carrier density in the QW, while the ISB transition frequency *ω*_0_ can be freely selected in the mid-infrared and terahertz ranges through the semiconductor heterostructure design. In this way, it is possible to obtain different regimes of the light–matter coupling in individual subwavelength-sized microcavities, often evaluated by the ratio $\eta =\frac{\Omega_{R}}{\omega_{0}}$ , spanning from the weak regime *η* < 0.05 to the (ultra)-strong regime for *η* > 0.1 [16]. The intriguing possibilities of controlling and manipulating these mixed light and matter quantum states paved the way for the development of novel devices such as polariton lasers, ultrafast modulators, and quantum information processors [15], [17].

Thanks to their inherent single-valued absorption spectrum and temperature independence, ISBTs in PQWs enclosed in an optical cavity has led to the realization of the ultra-strong light–matter coupling at room temperature and at THz frequencies [18]. Notably, all reported studies on PQWs are based on III–V compound semiconductors, in which the parabolic potential has been achieved first by digitally alloying GaAs and Al_0.15_Ga_0.85_As layers [11], and, only in the last few years, by implementing continuously graded Al_1−*x*_Ga_*x*_As quantum wells [19].

In recent years, group-IV QWs have emerged as a promising alternative to the III–V ones, offering a material platform for advanced electronic, photonic, and quantum functionalities, compatible with the well-established silicon technology base. Various applications ranging from photonics [20] to quantum computing [21] and microelectronics [22] have been proposed. In this context, Ge-rich Si_1−*x*_Ge_*x*_ QWs (*x* > 0.80) formed in the conduction band of n-type multilayers are particularly promising for the development of photonic devices operating in the THz range thanks to their relatively low confinement mass (∼0.13 *m*_0_) and simpler subband structure with respect to p-type SiGe systems [23], [24].

These characteristics suggest that n-type Ge-rich SiGe PQWs embedded into microcavities may also be used to achieve the strong coupling regime at THz frequencies also in group IV based structures, provided that high-quality samples can be satisfactorily realized with the existing epitaxial techniques. As a matter of fact, the epitaxial growth of n-type high Ge content SiGe QWs on Si substrates is more challenging than that of their III–V counterparts, mainly because of the large (4.2 %) mismatch existing between the Ge and Si lattice parameter, eventually leading to the plastic relaxation of the QW structures if not correctly managed. Furthermore, the control over the QW compositional profile is complicated by the tendency of Ge atoms to segregate on top of the growing surface and by the Si–Ge intermixing effect occurring during the growth. Also the realization of a sharp doping profile is problematic, due to the well-known tendency of donor atoms to “float” on Ge during the growth [25]. Indeed, only the recent improvements in the heteroepitaxial process of this material system have allowed a high degree of control over these physical effects [26], [27], [28], as witness by the observation in 2021 of ISB electroluminescence in the THz range from n-type Ge/SiGe QCL structures [29].

In view of the above observations, the demonstration of high-quality SiGe based PQWs represents another important milestone toward the full exploitation of this technologically relevant material system. A first attempt in this direction has been made in Ref. [30] where the authors reported on a set of Ge-rich SiGe PQWs. However, the investigated samples were undoped and the structural data highlighted strong deviations from the target parabolic profile. Genuine parabolic confining potential in n-type SiGe multilayer systems have been obtained only in 2021, using the same reactor here adopted: we have presented the experimental evidence of conduction-band intersubband transition energies around 20 meV, resulting from compositional graded Si_1−*x*_Ge_*x*_ parabolic quantum wells [31]. To consolidate and extend this result, here we report on a comprehensive study of the structural and optical properties of compositional graded Si_1−*x*_Ge_*x*_ PQWs having different well width and doping levels. The experimental features have been benchmarked against numerical ISB absorption data obtained by a multivalley self-consistent Schrödinger–Poisson solver. To highlight the unique properties of the harmonic confining potential, we have here designed PQW profiles corresponding to lower energy ISBTs with respect to those of Ref. [31], thus targeting an energy range down to 10 meV where the ISB spectral properties of square QWs are more sensible to thermal effects.

Our results demonstrate that UHV-CVD permits the realization of a stack of identical compositional graded Si_1−*x*_Ge_*x*_ PQWs with high reproducibility of the growth along the stack and in different samples. The high quality of these samples allowed the observation of narrow ISB absorption peaks and the tuning of the ISB absorption energy in the range between 10 and 20 meV (2.5–5 THz). Due to their parabolic confining potential, the measured absorption energy results to be temperature independent and rescales linearly with the inverse of the well width *W*.

These achievements are promising for the perspective use of compositional graded Si_1−*x*_Ge_*x*_ PQWs for the development of CMOS compatible novel optoelectronic devices operating in the THz range.

## 2 Methods

Samples were grown by means of UHV-CVD in a cold-wall reactor employing ultrapure germane and silane without the use of carrier gases at a pressure *p* ∼ 1 mTorr. The parabolic compositional profile Si_1−*x*_Ge_1−*x*_ with *x* in the 0.8–1 range is obtained by keeping the GeH_4_ gas flow constant and gradually varying the SiH_4_ flow by means of calibrated mass flow controllers (the SiH_4_/GeH4 flux ratio *R*_flux_ have been varied from *R*_flux_ = 0 during the pure Ge deposition to *R*_flux_ = 1.24 for *x* = 0.82). The sample active region typically consisted of alternating several identical PQWs and SiGe barriers. The growth temperature for this active stack was set at 460° and the average growth rate was 6 nm/min. N-type modulation doping was achieved by codepositing phosphine to a thickness of 3–5 nm in the center of the SiGe barriers.

The active layer stack was deposited on top of a reverse graded (RG) virtual substrate (VS), consisting of a relaxed Ge film grown directly on the Si substrate using a multi-temperature approach, and a relaxed Si_1−*y*_Ge_*y*_ buffer layer [32]. The final composition y of the SiGe buffer matched the average composition of the active stack to ensure the strain compensation condition and avoid the plastic relaxation of the active region.

The structural characterization of the samples was conducted using high resolution scanning transmission electron microscopy (STEM), X-ray diffraction (XRD), and time-of-flight secondary ion mass spectroscopy (ToF-SIMS). STEM measurements have been realized with a FEI Titan microscope (FEI Company, Hillsboro, Oregon, USA) operating at 200 kV. It was equipped with aberration-corrected magnetic lenses to obtain electron probes with a diameter in the range of 1–2 Å and beam currents of 200 pA. A CEOS CESCOR corrector was used to achieve a resolution of 0.8 Å. Images were recorded using a high-angle annular dark field (HAADF) detector (FEI Company, Hillsboro, Oregon, USA). XRD measurements were performed at room temperature using a Rigaku SmartLab instrument with a rotating anode and line-focus geometry, featuring a Ge(400) × 2 channel-cut beam collimator. Tof-SIMS compositional profiles were acquired in a ToF-SIMS V from IONTOF. The depth profiles were acquired using 0.5–1 keV a Cs^+^ ion beam.

Fourier-transform infrared (FTIR) spectroscopy was performed in a side-illuminated single-pass waveguide configuration with a Bruker Vertex 70v spectrometer (Bruker, Ettlingen, Germany) equipped with a helium-flow cryostat (Janis Research company, Woburn, Massachusetts (USA)). The lateral facets of our 2.5 mm long samples were cut at a 70° angle relative to the growth plane, and the top surface close to the QW stack was coated with a metal bilayer (Ti/Au 10 nm/80 nm) [25], to align the electric field of the radiation propagating through the active region almost parallel to the ISB dipole moment (i.e., TM polarized). The dichroic transmission spectra *T*(*ω*) = *T*_TM_(*ω*)/*T*_TE_(*ω*) were measured, ensuring that polarization-independent spectral features unrelated to ISB transitions were suppressed.

The electronic band structure, electron wave-functions, and ISBT absorption spectra of the investigated samples have been calculated self-consistently, relying on a multivalley effective mass Schrödinger–Poisson solver. A detailed description of the model can be found in Ref. [33].

## 3 Results and discussion

### 3.1 Sample design and structural characterization

**Figure 1:**
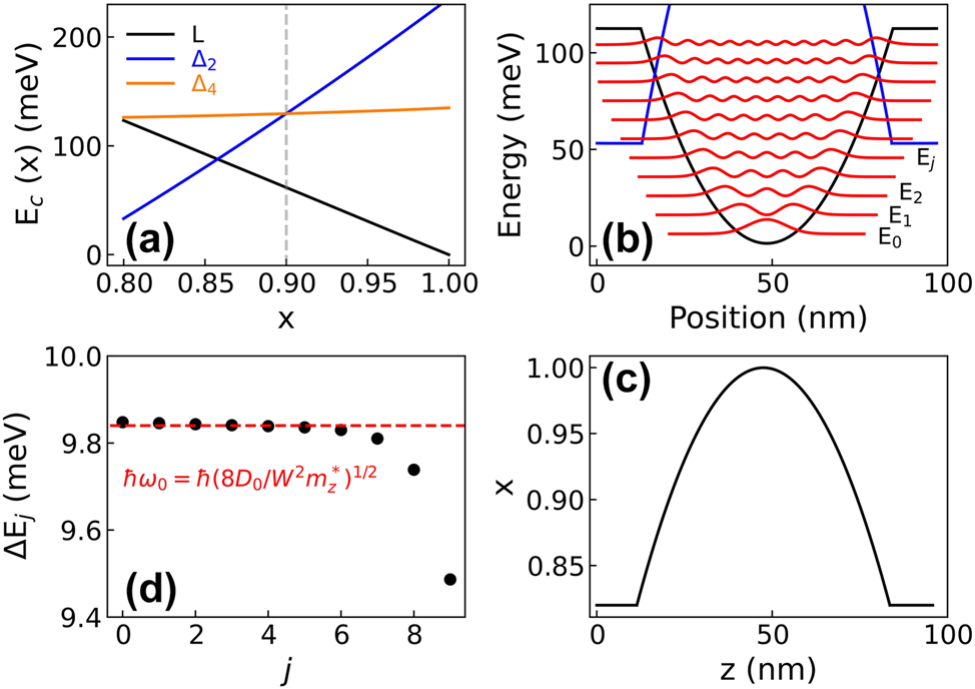
Bandstructure calculations. (a) Conduction band energy at the *L*, Δ_2_, and Δ_4_ valley edges as a function of the Ge content *x* in the alloy at 10 K. The energies are referred to the $E_{c}^{L}\left(1\right)$ value, set to 0. In the calculation, the in-plane lattice parameter was set equal to that of a relaxed Si_0.1_ Ge_0.9_ VS to account for the need of a strain compensation strategy when growing Ge-rich multi-quantum well samples. In this condition, the Si_1−*x*_ Ge_*x*_ alloy is tensile strained for *x* < 0.9 and compressively strained for *x* > 0.9. Notice that the in-plane tensile strain shifts upward the Δ_2_ edge with respect to the Δ_4_ one, while the opposite holds for compressive strain. Conversely, the energy of *L* valleys, being controlled by the hydrostatic component of the strain tensor only, keeps their fourfold degeneracy. (b) Potential *V*(*z*) = $E_{c}^{L}\left(x\left(z\right)\right)$ (black line) for a PQW having the graded compositional profile shown in panel (c); energy levels *E*_*j*_ and squared wavefunctions of the quantized states (red lines) are also reported. The edge profile at Δ_2_, $E_{c}^{\Delta_{2}}\left(x\left(z\right)\right)$ (blue line) is also displayed. (c) Ge content *x* in a PQW as a function of the position along the growth direction *z*. (d) Energy spacing of subsequent levels Δ*E*_*j*_ = *E*_*j*+1_ − *E*_*j*_.

In biaxially strained high Ge content Si_1−*x*_ Ge_*x*_ alloys (*x* > 0.8), *L*, Δ_2_, and Δ_4_ conduction valley minima feature very similar energies whose precise value depends on the Ge content *x* and on the strain status [34]. By using a multivalley self-consistent code in the parabolic *k*·*p* envelope function approximation, we have calculated the three conduction band edges $E_{c}^{L,\Delta_{2},\Delta_{4}}\left(x\right)$ as a function of the Ge content *x* in the 0.8–1 range, assuming that the in-plane lattice parameter is coherent to that of a relaxed Si_0.1_ Ge_0.9_ VS to account for the need of a strain compensation strategy when growing Ge-rich multi-quantum well samples. In Figure 1(a), the *L* and Δ point band edges as a function of *x* in the Ge-rich alloys are reported showing that for *x* > 0.85 the conduction band minimum is at *L*. Consequently, in compositional graded Ge-rich Si_1−*x*_ Ge_*x*_ quantum wells where the Ge content *x* = *x*(*z*) is changed along the growth direction *z*, the potential *V*(z) felt by electrons in the well is determined by the *L* conduction minimum profile $E_{c}^{L}\left(x\left(z\right)\right)$ [34]. The maximum achievable band-offset at *L* in the range *x* = [0.8, 1] is 126 meV and in this range $E_{c}^{L}\left(x\right)$ exhibits a linear behavior. We point out that in our modeling, $E_{c}^{L}\left(x\right)$ includes nonlinear terms, quadratic contributions being for instance related to the product of the L-point deformation potential with the strain field, since both these two quantities depend linearly on *x*. Nevertheless, Figure 1(a) indicates that those terms are practically negligible. This is confirmed by the inspection of Figure 1(b) where the potential *V*(*z*) = $E_{c}^{L}\left(x\left(z\right)\right)$ calculated assuming the parabolic compositional profile of Figure 1(c) (well width *W* = 72 nm and Si_0.18_Ge_0.82_ barrier layers as in most of our samples) features a quadratic behavior with a depth *D*_0_ = 112 meV. It follows, as highlighted in Figure 1(d), that the calculated energy spacing of subsequent levels Δ*E*_*j*_ = *E*_*j*_ − *E*_*j*−1_ is practically constant up to *j* = 7, in good agreement with the spacing expected in an infinite parabolic potential *ℏω*_0_ = *ℏ*(8*D*_0_/*W* ^2^ *m*_z_^*^)^1/2^ where *m*_z_^*^ = 0.13*m*_0_ is the electron effective confining mass along the growth direction *z*. As a final remark, Figure 1(b) shows that the Δ_2_ edge profile features a reversed parabolic shape with a minimum in the barrier region where the relative Δ_2_ subband states are confined. We point out that in Ge-rich doped QWs, the coupling of Δ_2_ states in the barriers with the populated *L* ones in the wells is negligible, as discussed in Ref. [35].

To investigate the properties of SiGe parabolic quantum wells, we have deposited a series of samples (see Table 1) having a parabolic compositional graded Si_1−*x*(*z*)_ Ge_*x*(*z*)_ profile (0.82 < *x* < 1), with different well width *W* and doping levels *n*_2D._

**Table 1:** Structural and optical parameters of the investigated PQW samples. The number *N*_QW_ of the deposited PQWs, the nominal values of the well width *W*, the multilayer stack period *p*_nom_, the Ge content *x*_bar_ in the barrier, and the electron sheet density *n*_2D_ as well as experimental values of the multilayer stack period *p*_XRD_ and the average Ge content *x*_av_^XRD^ in the active region as obtained by the XRD data are reported. Experimental values of the absorption energy *ℏω*_*abs*_, the FWHM of the absorption peak, and the sheet density *n*_2D_^*^ as evaluated from the FTIR data acquired at 100 K are also stated.

Sample #	*N*_QW_	*W* (nm)	*p*_nom_ (nm)	*x*_bar_ (%)	*n*_2D_ (cm^−2^)	*P*_XRD_ (nm)	*x*_av_^XRD^ (%)	*ℏω*_abs_ (meV)	FWHM (meV)	*n*_2D_^*^ (cm^−2^)
2452	21	76	90	82	–	88	92.0			
2454	21	74	92	82	2.5 × 10^11^	93	91.8			
2456	21	74	92	82	2.5 × 10^11^	91	91.7			
2455	21	74	92	82	2.5 × 10^11^	92	91.8	10.0	3.0	1.5 × 10^11^
2458	21	72	91	82	2.5 × 10^11^	90	91.7	10.7	2.1	1.2 × 10^11^
2459	21	72	91	82	5 × 10^11^	91	91.5	11.8	5.0	3 × 10^11^
2460	25	61	76	82	3.7 × 10^11^	79	91.2	13.2	5.0	2.8 × 10^11^
2354	20	46	62	82	8 × 10^11^	62	91.2	19.0	6.2	3.5 × 10^11^
2433	25	66	77	84	2.5 × 10^11^	78	92.7	10.7	2.3	1.5 × 10^11^
2420	25	66	77	84	5 × 10^11^	76	92.9	11.8	5.5	3 × 10^11^

The well width *W* has been varied in the range 76–46 nm to achieve transition energies in the range 10–20 meV (2.5–5 THz). We deposited a stack of *N*_QW_ = 20–25 identical wells for an overall thickness of the active region of ∼1.5–2 μm. These values have been selected envisioning the embedding of the parabolic QW stacks into microcavities to test the strong coupling limit [36]. As for the doping, we varied the phosphorus concentration in the central part of the barrier in order to tune the electron sheet densities *n*_2D_ in the [1 × 10^11^ − 5 × 10^11^] cm^−2^ range.

**Figure 2:**
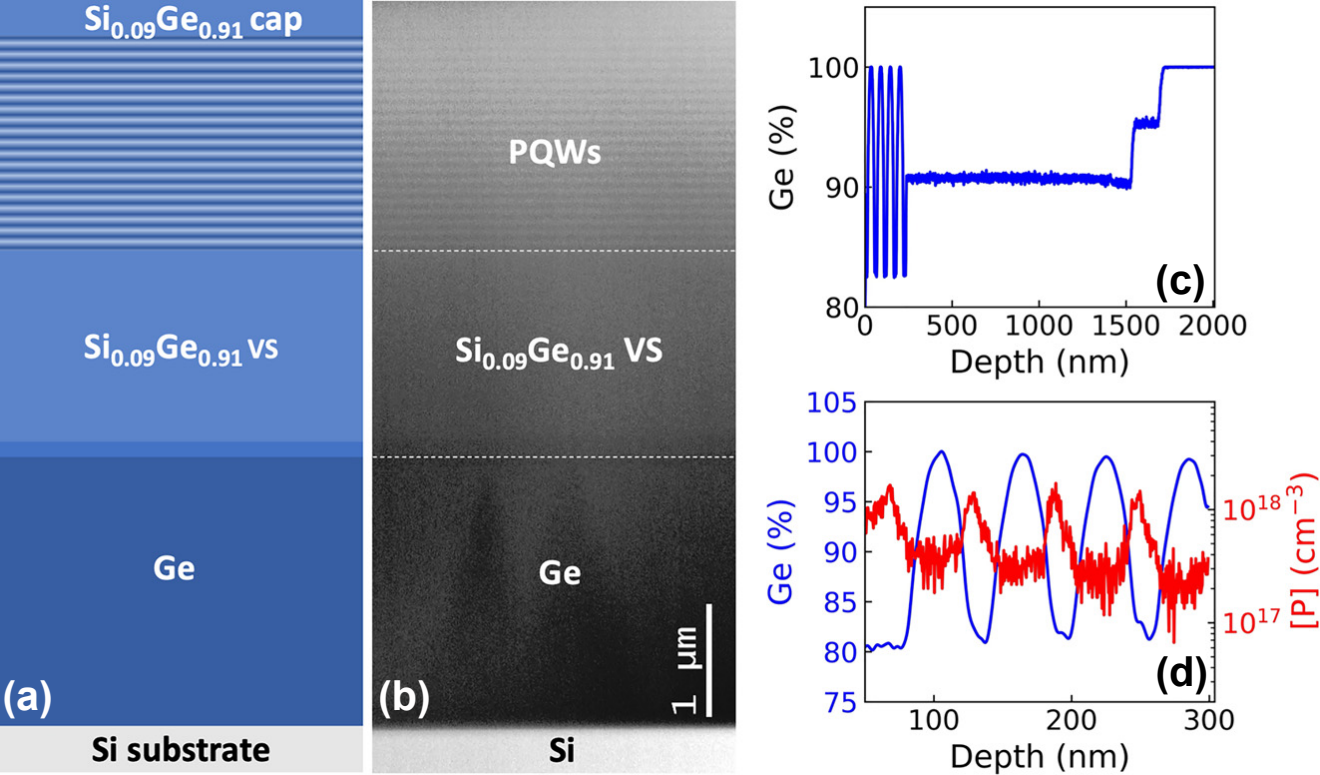
Structural characterization. (a) Sketch of the deposited multilayered structure. (b) STEM image of the 2452 PQW sample. (c) SIMS Ge content profile showing the RG VS region and the first 4 PQWs of the stack. (d) SIMS *P* and Ge content profiles of 4 periods acquired on a PQW sample with *p*_nom_ = 60 nm. The central 5 nm of the SiGe barriers are doped with a nominal *P* concentration *N*_3D_ = 1 × 10^18^ cm^−3^.

The complete multilayered structure deposited on the Si(001) substrate is sketched in Figure 2(a) and the STEM image acquired on sample 2452 is shown in Figure 2(b). A reverse graded SiGe VS with a final Ge content *y* = 0.91 has been deposited in all the samples. To reach this final Ge content *y*, two 150 nm thick layers with increasing Si content have been deposited on the Ge buffer layer, as evidenced by the SIMS Ge content profile of the graded region of the virtual substrate and of the first 4 PQWs reported in Figure 2(c). To fulfill the strain-compensation conditions, the thickness of the SiGe barriers in the active region has been varied in order to maintain the average Ge content *x*_av_ close to 0.91. In our growth conditions, misfit and threading dislocations resulting from plastic relaxation of the Ge buffer and of the SiGe layers are mostly confined in the bottom part of the RG-VS, i.e., in its graded region [27]. The threading dislocation density in the topmost part of the sample has been quantified by Secco etching pit count measurements performed on similar samples [27], being ∼3 × 10^6^ cm^−2^, a state of the art value for high Ge content SiGe heterostructures deposited on Si substrates.

In Figure 2(d), the *P* and Ge SIMS content profiles of 4 periods of the active region of a sample featuring *p*_nom_ = 60 nm and nominally doped with a concentration of phosphorus atoms *N*_3D_ = 1 × 10^18^ cm^−3^ in the central 5 nm of the SiGe barriers are reported. The measurements demonstrate that in our growth conditions dopants remain well confined in the barriers with only a slight shift upwards of the *P* distribution with respect to the barrier center. This is a very important and not trivial achievement due to the *P* atoms tendency to float on Ge during the growth that could have conflicted with the need of confining donor atoms in the barriers to exploit the unique characteristic of the compensation of electron correlation effects in parabolic QWs.

**Figure 3:**
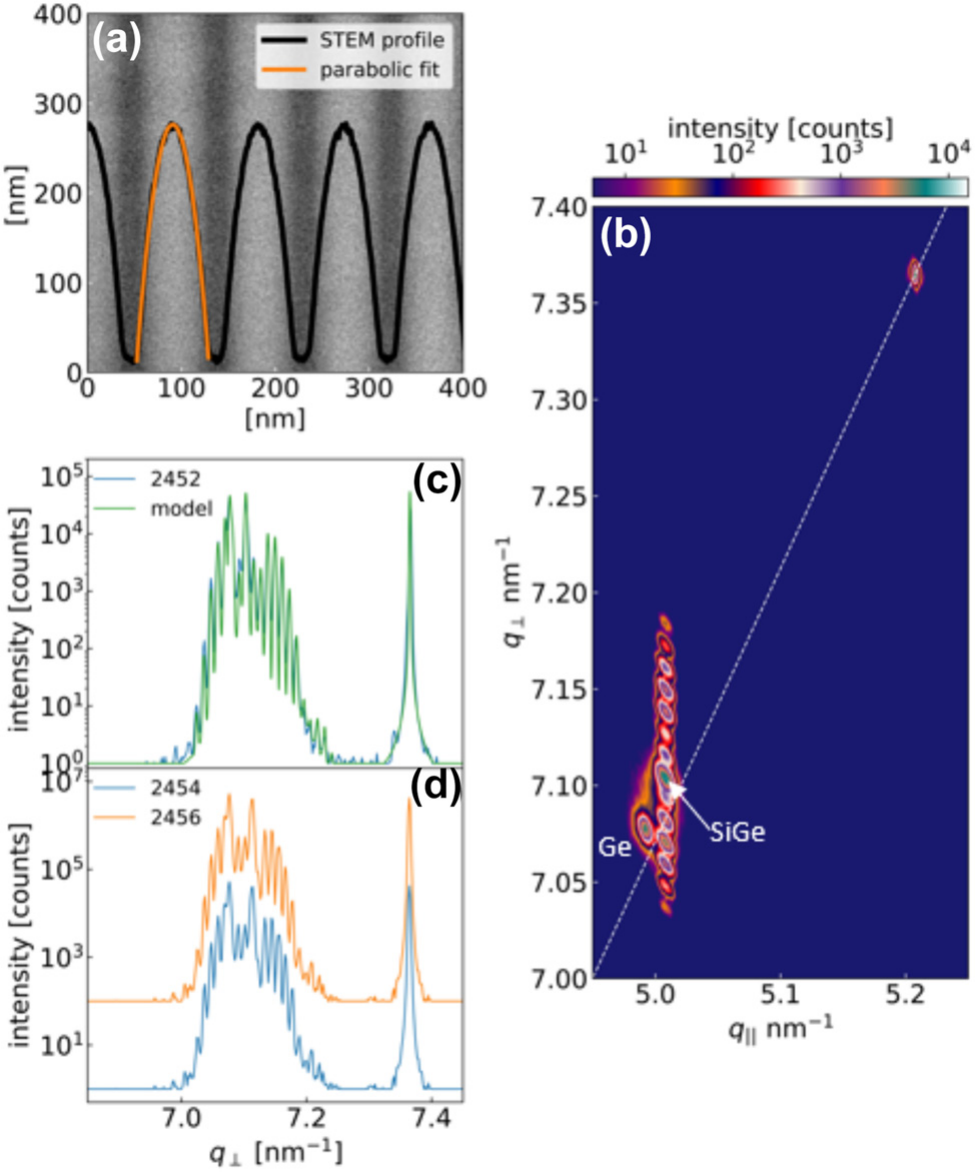
Measure of parabolic concentration profile. (a) STEM image of sample 2452. The intensity profile and its parabolic fit are superimposed on the image. (b) Reciprocal space map of asymmetric 224 reflections of sample 2452. The dashed white line represents the reference values for relaxed Si_1−*x*_Ge_*x*_ alloys. (c) 004 XRD *ϑ*/2*ϑ* curve of sample 2452 (blue curve) and dynamical simulation of the curve (green line) calculated assuming the well compositional profile resulting from the parabolic fit of the STEM data reported in (a). (d) 004 XRD rocking curves of samples 2454 and 2456 deposited using identical conditions.

The structural characterization of the samples has been performed by means of high-resolution STEM and XRD measurements. The data acquired on sample 2452, having a nominal period in the multi-quantum well stack *p*_nom_ = 90 nm, are reported in Figure 3. In panel (a), the STEM image of 5 periods of the active region of the sample is shown. The resulting intensity profile is in excellent agreement with the parabolic fit, demonstrating the capability of our UHV-CVD reactor in controlling at the nm scale the continuous grading of the SiGe alloy compositional profile. This achievement represents a significant step forward with respect to the previous attempt of growing graded Si_1−*x*_Ge_*x*_ PQW samples by plasma-enhanced CVD reported in Ref. [30]. The 224 XRD reciprocal space map around asymmetric reflections of the layers is displayed in Figure 3(b). The positions of the VS spots associated to the Ge and the Si_0.09_Ge_0.91_ layers reveal the presence at room temperature of a tensile strain of about +0.15 %, which can be ascribed to the difference between the coefficients of thermal expansion in Ge and Si [37], [38]. Multiple orders of superlattice (SL) satellites, resulting from the PQW periodicity, that are perfectly vertically aligned to the Si0_0.09_Ge_0.91_ spot are present in the map, indicating that the entire PQW stack is coherent with the in-plane lattice parameter of the underlying VS. In Figure 3(c), we show the XRD *ϑ*/2*ϑ* curves around the 004 Ge and 004 Si Bragg peaks together with a simulation curve calculated using the parabolic fit of the STEM image as input for the well compositional profile. The sharp superlattice peaks confirm the high crystalline quality and the regular periodicity of the PQWs in the stack. Moreover, the good agreement between XRD data and simulations confirms the parabolic symmetry of the compositional profile over a relatively large area of the sample (∼4 mm^2^). Finally, the likeness of the XRD *ϑ*/2*ϑ* curves acquired on two nominally identical samples and reported in Figure 3(d) demonstrates the reproducibility in our reactor of the growth of different samples.

The MQW stack period and the average Ge content in the stack measured by XRD on all the investigated samples are reported in Table 1. A very good agreement with respect to the nominal values has been found demonstrating once more the control acquired in the deposition process.

### 3.2 Optical properties of doped Si_1−*x*_Ge_*x*_ PQWs

**Figure 4:**
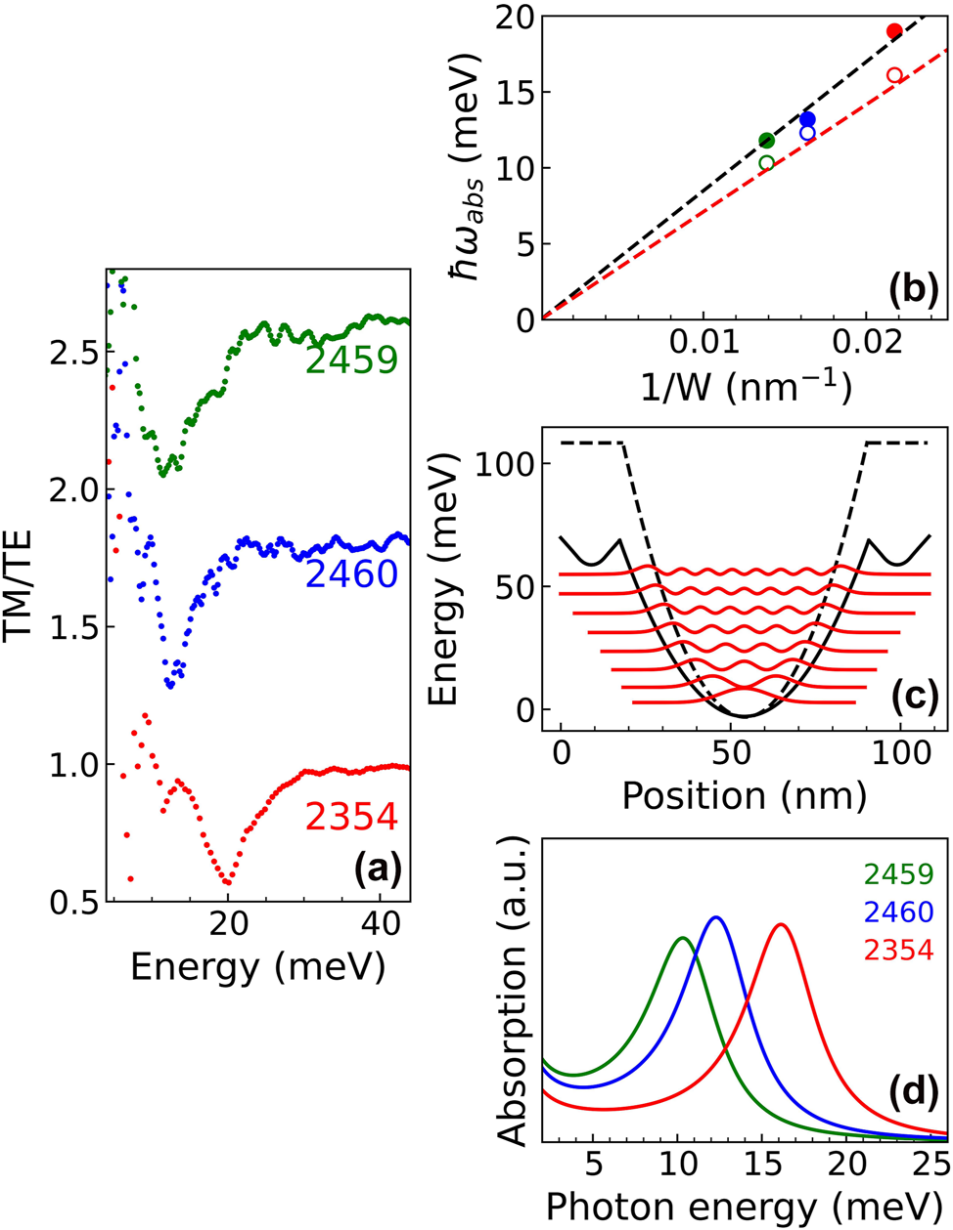
Terahertz spectroscopy measurements. (a) Dichroic transmission spectra acquired at 100 K of samples 2459, 2460, and 2354 having an electron sheet density ∼3 × 10^11^ cm^−2^ and well width 72 nm, 61 nm, and 46 nm, respectively. (b) Experimental (full circles) and theoretical (open circles) absorption energies as a function of *W* ^−1^. Black dashed line represents the linear fit of the experimental data and the red dashed line the parabolic bare energy behavior *ℏω*_*0*_(*W*) = *ℏ*(8**D**_**0**_/*m*_z_*)^1/2^/*W*. (c) Potential *V*(*z*) (continuous black line) for sample 2459 along the growth direction; energy levels *E*_*j*_ and squared wavefunctions of the quantized states are also reported; the potential calculated for an undoped PQWs with the same shape is displayed as a dashed black line. The two potential curves have been aligned in energy at their minimum: (d) Theoretical absorption spectra for the samples in panel (a).

Intersubband transition absorption spectra have been measured on a subset of the doped samples of Table 1. The linear dichroic spectra have been acquired at different temperatures in the energy range where intersubband transitions are expected from numerical calculations. Although in this region several absorption lines are observed in the *TM* and *TE* spectra, the dichroic transmission signal is characterized in all the investigated samples by a single pronounced dip, which is due to a reduced transmission of the TM mode and which monotonically blue-shifts upon decreasing the well width (see Figure 4). Following the procedure reported in Ref. [33], the dimensionless single well absorption coefficient *α*_2D_(*E*) has been determined and the central absorption energy *ℏω*_*abs*_, the full width half maximum FWHM of the absorption peak, and the electron sheet density *n*_2D_* are extracted by fitting the resulting *α*_2D_(*E*) spectrum with a Lorentzian curve. The values estimated from the experimental data acquired at 100 K on all the measured samples are reported in Table 1.

We point out that the absolute values of both nominal (*n*_2D_) and measured (*n*_2D_*) sheet densities are affected by a relative error of ∼30 %, due to the difficulties of a precise calibration of the *N*_3D_ phosphorus concentration confined in very thin doped layers, and to the uncertainty in the evaluation of the single well absorbance from the FTIR dichroic spectra acquired in multilayered samples with the side-illuminated single-pass waveguide configuration [1]. However, since the same doping procedure and FTIR data analysis have been used systematically, the *relative* variations of both *n*_2D_ and *n*_2D_* among different samples are much more accurate. Despite these uncertainties, the fact that *n*_2D_* values are always lower than the *n*_2D_ ones may be due to the small energy difference between donor levels originating from the Δ_2_ edge states confined in the tensile strained SiGe barriers and the fundamental *L* subband state in the well, which can limit the effectiveness of the transfer doping mechanism [33]. Interestingly, the largest difference between *n*_2D_ and *n*_2D_* is observed in sample 2354 which, having the thickest barrier, features the lowest energy difference between the fundamental *L* subband state and the Δ_2_ donor level.

The low temperature dichroic transmission spectra of samples 2459, 2460, and 2354 having different well width and comparable electron sheet densities in the well *n*_2D_* ∼ 3 × 10^11^ cm^−2^ are reported in Figure 4(a). As expected, the transmission dip energy blue-shifts by decreasing the well width *W*. As shown in Figure 4(b), the dependence of the measured absorption energy *hω*_abs_ (full circles) on 1/*W* follows the typical linear behavior foreseen for electron ISBTs in a harmonic potential. The electronic band structure and the absorption spectra of the samples have been calculated self-consistently. In modulation doped samples, the electrostatic (Hartree) charge effects influence the *L* band profile decreasing the depth of the effective potential felt by electrons as shown in Figure 4(c), where the effective potential profile, the electron energy levels, and the square modulus of the wavefunctions calculated for sample 2459 at 10 K are reported. The bare potential calculated for an undoped PQW with the same compositional profile is also reported for comparison. The simulated absorption spectra of the three samples are displayed in Figure 4(d). Estimated absorption energies in Figure 4(b) (open symbol) have been calculated correcting the subband transition energy, obtained considering the Hartree potential, to account for the depolarization shift effect, following Ref. [39]. Minor contributions stemming from band nonparabolicity and other many-body effects have been neglected. The shrinking of the energy level separation due to the Hartree potential is compensated by the depolarization shift so that the calculated absorption energies result in good agreement with the bare harmonic energy *ℏω*_*0*_ (red dashed line). However, the theoretical energies are slightly smaller with respect to the experimental data suggesting that the ratio *D*_0_/*m*_*z*_ could be slightly underestimated in our simulations.

**Figure 5:**
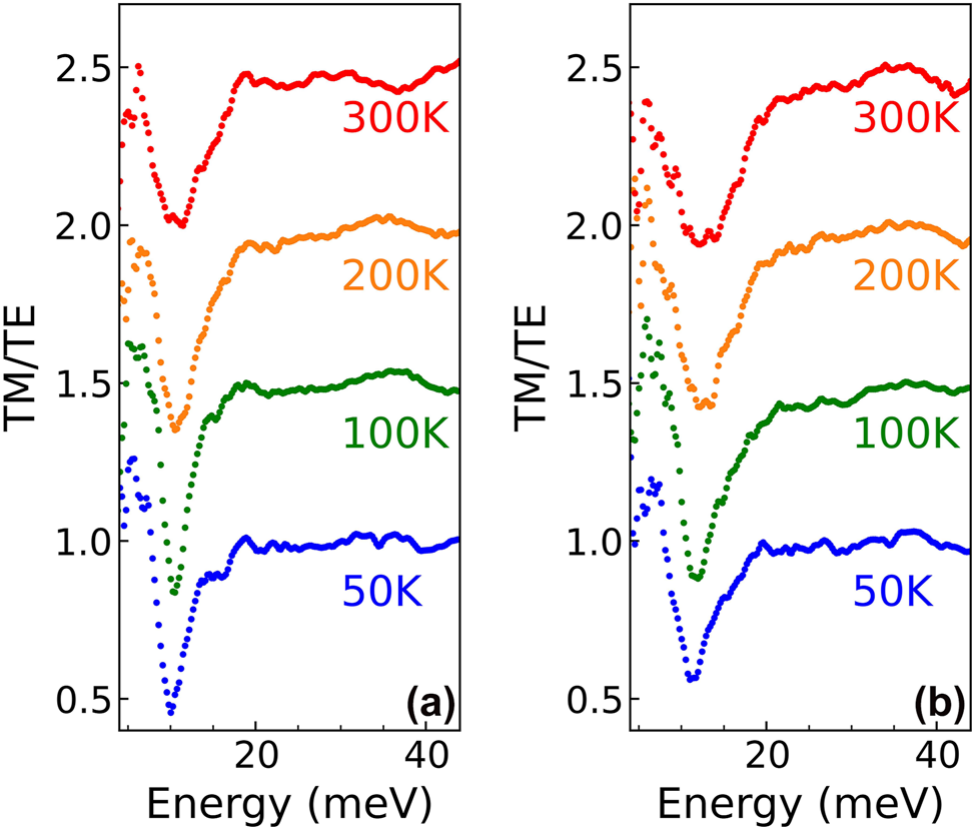
Dichroic transmission spectra acquired at different temperatures for two samples with the same well profile but different doping. (a) Sample 2433 with *n*_2D_* ∼ 1.5 × 10^11^ cm^−2^. (b) Sample 2420 with *n*_2D_* ∼3 × 10^11^ cm^−2^.

In Figure 5 the dichroic transmission spectra as a function of temperature of samples 2433 and 2420 having the same well profile but different doping ((a) *n*_2D_* ∼ 1.5 × 10^11^ cm^−2^, (b) *n*_2D_* ∼ 3 × 10^11^ cm^−2^) are reported. The transmission dips in these two samples are practically at same energy, the one featuring the larger carrier density being blueshift by 1 meV only. Remarkably, we find that in each sample the absorption energy is almost temperature independent despite in this THz range the subband population strongly depends on *T*. As already pointed out, this is a unique “fingerprint” of parabolic quantum wells, whose absorption energy is independent on the distribution of electron in subbands. This contrasts with terahertz ISB absorption in square QWs, where the lineshape and peak absorption energy vary with temperature [40]. Consistently, we have observed a significant variation of the ISB absorption spectrum with temperature when the PQWs is directly doped in the well, since in this case the cancellation of electron correlation effects does not occur [31].

The main difference between the spectra of the two samples of Figure 5 is the increased linewidth observed at higher doping: in sample 2433 (*n*_2D_* ∼ 1.5 × 10^11^ cm^−2^), a narrow FWHM of about 2.3 meV has been measured at 100 K, increasing to 5 meV at 300 K. On the contrary, sample 2420 (*n*_2D_* ∼ 3 × 10^11^ cm^−2^) shows a FWHM ∼ 5.5 meV, almost constant with temperature. As shown in Table 1 where the FWHM at 100 K of all the investigated samples are reported, FWHMs ∼ 2 meV and ∼ 5 meV are typical values for these doping levels, independently on the well width and absorption energy. The observed increase of the linewidth as a function of the electron sheet density is in good agreement with previous results obtained in rectangular quantum wells. Interestingly, we notice that the values here reported at low doping are sensitively smaller with respect to those previously measured in rectangular Ge/SiGe QWs [41] typically featuring FWHM slightly larger than 4 meV. This effect may be attributed to a reduction of the scattering associated to the interface roughness occurring in systems without sharp interfaces.

The linewidth analysis, we performed with square QWs samples in [41] highlighted the presence of a non-negligible contribution of about 1–2 meV to the total width, attributed to structural crystal defects brought about by the fraction of treading defects which, overcoming the RG-VS region, penetrates in the QWs one. Although the crystalline quality of the SiGe heteroepitaxy in our reactor has been significantly improved in the last few years [27], the same mechanism can affect the lineshape of the PQW spectra here investigated, resulting in larger low temperature FWHM values with respect to III–V ones [19]. Considering the superior capability of achieving a sharp delta-like donor profile in III–V heteroepitaxy, also the spatial broadening of the *P* concentration along the growth direction, associated to the surface kinetics of donor atoms in the SiGe environment, can contribute to the enlargement of the absorption peak. We point out here that, in order to achieve a high filling factor of quantum wells in the stack, barrier thicknesses <18 nm have been deposited in our samples. We believe that the narrowness of the absorption resonance of Si_1−*x*_Ge_*x*_ PQW samples could be improved by depositing thicker barriers and decreasing the growth temperature during the phosphine codeposition step, so to increase the spacer layer thickness between the doped region and the well [40]. Finally, we notice that in III–V QWs, a larger temperature dependence of the linewidth has been observed, so that the two material systems exhibit more similar value of the FWHM/*E*_abs_ ratio at room temperature [31].

The absorption spectra of our PQWs represent a promising starting point to achieve and investigate strong radiation-matter coupling in this technologically relevant material system. Following Ref. [8], [42], [43], in a photon cavity of thickness *L*_cav_ with a stack of *N*_QW_ PQWs doped with an electron sheet density *n*_2D_, the intersubband polariton energy splitting *E*_up-down_ at the matching condition *ω*_0_ = *ω*_*cav*_ is:

$$E_{\text{up }-\text{ down}}==2\hbar \Omega_{R}=\hbar \sqrt{\frac{e^{2}fN_{QWn_{2D}}}{\varepsilon \varepsilon_{0}m_{z}^{*}L_{\text{cav}}}}.$$

Therefore, using an oscillator strength value *f* = 1, *L*_cav_ = 2 μm and *N*_QW_ = 25, *n*_2D_ = 2 × 10^11^ cm^−2^, as in our samples, we estimate an ISB polariton energy splitting *E*_up-down_ ∼ 5 meV and a strength ratio $\eta =\frac{\Omega_{R}}{\omega_{0}}$ ∼ 0.2 at 2.5 THz, well within the ultra-strong coupling regime. This estimated splitting value, which is larger than the measured FWHM, make feasible the future demonstration of ISB polaritons in Ge-rich PQWs.

Different approaches have been pursued to realize optical cavities aimed at the observation of the strong coupling regime with ISBTs. Most of them rely on lithographic patterning of metal structures directly on the epitaxial wafer, with or without etching the surrounding material. It is well known that the most important constraint in the comparison of different cavity designs is related to the dipole orientation, which requires a radiative electric field with polarization orthogonal to the quantum well plane. A second important parameter is the cavity volume that must be small, possibly subwavelength sized. To meet these requirements, the active region is usually placed within nondispersive metal–insulator–metal (MIM) microresonators, which are typically fabricated with the substrate-transfer technique. Among them, we mention the one-dimensional periodic grating [36], the capacitor-inductor cavities of Ref. [11], [18], [44], and the patch-antenna cavity discussed in Ref. [43], where the extension of the cavity in the vertical direction is accompanied by a small volume and a totally vertical and homogeneous electric field. The implementation of the substrate transfer technique is more complicated in SiGe epitaxial layers with respect to III–V materials, because of the difficulties related to the material-selective etching processes and owing to the lattice strain (thermal and epitaxial) featured by SiGe heterostructures. Planar microcavities relying, e.g., on deep-etched split-ring resonators, previously realized for III–V materials [45], are not efficient in the SiGe case due to the presence of the thick Ge virtual substrate with high dielectric constant that traps most of the electromagnetic energy density in an inactive layer. The solution may come again from epitaxy: while noble metals such as gold or aluminum have almost always been used for the cavity mirrors, in the THz range it is also possible to employ epitaxially grown, heavily doped degenerate semiconductors as substitutes for the metal layers. Indeed, the complex dielectric function of n-Ge with *n* > 10^19^ cm^−3^, measured in [46], indicates that a reflectivity *R* ∼ 1 can be achieved up to the mid-IR. For example, a plasma edge at 30 THz has been observed in epitaxial n-Ge with activated electron densities ∼2 × 10^19^ cm^−3^ and used to produce all-semiconductor nanoantennas [47]. We thus suggest that THz ISB polariton with SiGe PQWs can be demonstrated avoiding the substrate-transfer procedure and leveraging on a plasmonic-conductor ground plane below the quantum well stack, realized P-doping at ∼2 × 10^19^ cm^−3^ the Si_0.09_Ge_0.91_ VS layer.

## 4 Conclusions

High quality n-doped Ge-rich Si_1−*x*_Ge_*x*_ graded PQW samples have been designed, grown, and structurally and optically characterized. The SIMS, XRD, and STEM analysis demonstrate the high degree of control at the nanometer scale of the employed UHV-CVD reactor over the deposited compositional profile and the high reproducibility of the growth over the whole active region of the multilayered structure.

The intersubband absorption spectra display a sharp single-value resonance in the 2.5–5 THz range, fairly independent from doping and temperature, and inversely proportional to the well width, as expected for an harmonic potential. The observed absorption features are in good agreement with numerical calculations, evidencing that the significative shrinking of the energy levels due to the Hartree potential is compensated mainly by the energy blue-shift induced by the depolarization effect. A coupling strength ratio $\eta =\frac{\Omega_{R}}{\omega_{0}}$ ∼ 0.2 at *ω*_0_ = 2.5 THz, i.e., well within the ultra-strong coupling regime, has been estimated. We thus believe that SiGe parabolic quantum well, combined with THz optical cavities, can enable the exploration of the ultra-strong light–matter coupling limit in a silicon-based, CMOS foundry-compatible material platform, possibly operating at room temperature, opening new routes for the exploitation of these exotic quantum states in novel mainstream electronic devices.

## References

[1] Helm M. (1999). Chapter 1 the basic physics of intersubband transitions. *Semicond. Semimetals*.

[2] Schneider H., Liu H. C. (2007). Quantum well infrared photodetectors: physics and applications. *Springer Series in Optical Sciences, No. 126*.

[3] Palaferri D. (2015). Patch antenna terahertz photodetectors. *Appl. Phys. Lett.*.

[4] Faist J., Capasso F., Sivco D. L., Sirtori C., Hutchinson A. L., Cho A. Y. (1994). Quantum cascade laser. *Science*.

[5] Vitiello M. S., Scalari G., Williams B., De Natale P. (2015). Quantum cascade lasers: 20 years of challenges. *Opt. Express*.

[6] Faist J. (2013). *Quantum Cascade Lasers*.

[7] Kohn W. (1961). Cyclotron resonance and de Haas-van Alphen oscillations of an interacting electron gas. *Phys. Rev.*.

[8] Ciuti C., Bastard G., Carusotto I. (2005). Quantum vacuum properties of the intersubband cavity polariton field. *Phys. Rev. B*.

[9] Dini D., Köhler R., Tredicucci A., Biasiol G., Sorba L. (2003). Microcavity polariton splitting of intersubband transitions. *Phys. Rev. Lett.*.

[10] Forn-Díaz P., Lamata L., Rico E., Kono J., Solano E. (2019). Ultrastrong coupling regimes of light-matter interaction. *Rev. Mod. Phys.*.

[11] Geiser M., Castellano F., Scalari G., Beck M., Nevou L., Faist J. (2012). Ultrastrong coupling regime and plasmon polaritons in parabolic semiconductor quantum wells. *Phys. Rev. Lett.*.

[12] Anappara A. A., Tredicucci A., Biasiol G., Sorba L. (2005). Electrical control of polariton coupling in intersubband microcavities. *Appl. Phys. Lett.*.

[13] Anappara A. A., Tredicucci A., Beltram F., Biasiol G., Sorba L. (2006). Tunnel-assisted manipulation of intersubband polaritons in asymmetric coupled quantum wells. *Appl. Phys. Lett.*.

[14] Rajabali S. (2022). An ultrastrongly coupled single terahertz meta-atom. *Nat. Commun*..

[15] Raab J. (2020). Ultrafast terahertz saturable absorbers using tailored intersubband polaritons. *Nat. Commun*..

[16] Malerba M. (2022). Detection of strong light–matter interaction in a single nanocavity with a thermal transducer. *ACS Nano*.

[17] Colombelli R., Manceau J.-M. (2015). Perspectives for intersubband polariton lasers. *Phys. Rev. X*.

[18] Goulain P., Deimert C., Jeannin M. (2023). THz ultra‐strong light–matter coupling up to 200 K with continuously‐graded parabolic quantum wells. *Adv. Opt. Mater.*.

[19] Deimert C. (2020). Realization of harmonic oscillator arrays with graded semiconductor quantum wells. *Phys. Rev. Lett.*.

[20] Fischer I. A. (2022). On-chip infrared photonics with Si-Ge-heterostructures: what is next?. *APL Photonics*.

[21] Scappucci G. (2020). The germanium quantum information route. *Nat. Rev. Mater.*.

[22] Bogumilowicz Y. (2004). High Ge content Si/SiGe heterostructures for microelectronics and optoelectronics purposes. *Proc. – Electrochem. Soc.*.

[23] Paul D. J. (2010). The progress towards terahertz quantum cascade lasers on silicon substrates: progress towards THz QCLs on Si substrates. *Laser Photonics Rev.*.

[24] Grange T. (2019). Room temperature operation of *n* -type Ge/SiGe terahertz quantum cascade lasers predicted by non-equilibrium Green’s functions. *Appl. Phys. Lett.*.

[25] Ismail K., Chu J. O., Saenger K. L., Meyerson B. S., Rausch W. (1994). Modulation-doped *n* -type Si/SiGe with inverted interface. *Appl. Phys. Lett.*.

[26] Ciano C. (2019). Control of electron-state coupling in asymmetric Ge/Si−Ge quantum wells. *Phys. Rev. Appl.*.

[27] Simola E. T. (2023). Subnanometer control of the heteroepitaxial growth of multimicrometer-thick Ge/Si-Ge quantum cascade structures. *Phys. Rev. Appl.*.

[28] Grange T. (2020). Atomic-scale insights into semiconductor heterostructures: from experimental three-dimensional analysis of the interface to a generalized theory of interfacial roughness scattering. *Phys. Rev. Appl.*.

[29] Stark D. (2021). THz intersubband electroluminescence from n-type Ge/SiGe quantum cascade structures. *Appl. Phys. Lett.*.

[30] Ballabio A. (2019). Ge/SiGe parabolic quantum wells. *J. Phys. D: Appl. Phys.*.

[31] Montanari M. (2021). THz intersubband absorption in n-type Si1− *x* Ge *x* parabolic quantum wells. *Appl. Phys. Lett.*.

[32] Capellini G. (2010). Strain relaxation in high Ge content SiGe layers deposited on Si. *J. Appl. Phys.*.

[33] Busby Y. (2010). Near- and far-infrared absorption and electronic structure of Ge-SiGe multiple quantum wells. *Phys. Rev. B*.

[34] Virgilio M., Grosso G. (2006). Type-I alignment and direct fundamental gap in SiGe based heterostructures. *J. Phys.: Condens. Matter*.

[35] Ortolani M. (2011). Long intersubband relaxation times in n-type germanium quantum Wells. *Appl. Phys. Lett.*.

[36] Todorov Y. (2010). Optical properties of metal-dielectric-metal microcavities in the THz frequency range. *Opt. Express*.

[37] Capellini G., De Seta M., Zaumseil P., Kozlowski G., Schroeder T. (2012). High temperature x ray diffraction measurements on Ge/Si(001) heterostructures: a study on the residual tensile strain. *J. Appl. Phys.*.

[38] Manganelli C. L. (2020). Temperature dependence of strain–phonon coefficient in epitaxial Ge/Si(001): a comprehensive analysis. *J. Raman Spectrosc.*.

[39] Chen W. P., Chen Y. J., Burstein E. (1976). Interface EM modes of a “surface quantized” plasma layer on a semiconductor surface. *Surf. Sci.*.

[40] De Seta M. (2012). Narrow intersubband transitions in n-type Ge/SiGe multi-quantum wells: control of the terahertz absorption energy trough the temperature dependent depolarization shift. *Nanotechnology*.

[41] Virgilio M., Sabbagh D., Ortolani M., Di Gaspare L., Capellini G., De Seta M. (2014). Physical mechanisms of intersubband-absorption linewidth broadening in s -Ge/SiGe quantum wells. *Phys. Rev. B*.

[42] Geiser M. (2010). Strong light-matter coupling at terahertz frequencies at room temperature in electronic LC resonators. *Appl. Phys. Lett.*.

[43] Todorov Y. (2012). Polaritonic spectroscopy of intersubband transitions. *Phys. Rev. B*.

[44] Masini L. (2017). Continuous-wave laser operation of a dipole antenna terahertz microresonator. *Light: Sci. Appl.*.

[45] Dietze D., Andrews A. M., Klang P., Strasser G., Unterrainer K. (2013). J. Darmo, “Ultrastrong coupling of intersubband plasmons and terahertz metamaterials. *Appl. Phys. Lett.*.

[46] Frigerio J. (2016). Tunability of the dielectric function of heavily doped germanium thin films for mid-infrared plasmonics. *Phys. Rev. B*.

[47] Baldassarre L. (2015). Midinfrared plasmon-enhanced spectroscopy with germanium antennas on silicon substrates. *Nano Lett*..

